# The effect of genetic variants of *SLC22A18* on proliferation, migration, and invasion of colon cancer cells

**DOI:** 10.1038/s41598-024-54658-w

**Published:** 2024-02-16

**Authors:** Hyo Sook Song, Seung Yeon Ha, Jin-Young Kim, Minsuk Kim, Ji Ha Choi

**Affiliations:** Department of Pharmacology, Inflammation-Cancer Microenvironment Research Center, College of Medicine, Ewha Womans University, 25 Magokdong-ro 2-gil, Gangseo-gu, Seoul, 07804 Republic of Korea

**Keywords:** Genetics, Molecular biology, Biomarkers, Molecular medicine

## Abstract

Solute carrier family (SLC) transporters are expressed in the digestive system and play important roles in maintaining physiological functions in the body. In addition, SLC transporters act as oncoproteins or tumor-suppressor proteins during the development, progression, and metastasis of various digestive system cancers. SLC22A18, a member of the *SLC22* gene family, is an orphan transporter with an unknown endogenous substrate. Previous study revealed that *SLC22A18* is downregulated in colorectal cancer tissues and that it acts as a suppressor in colorectal cancer, although the effects of *SLC22A18* variants on colon cancer cell proliferation, migration, and invasion are unknown. Therefore, in this study, we identified *SLC22A18* variants found in multiple populations by searching public databases and determined the in vitro effects of these missense variations on transporter expression and cancer progression. Our results indicated that three missense *SLC22A18* variants—p.Ala6Thr, p.Arg12Gln, and p.Arg86His—had significantly lower cell expression than the wild type, possibly owing to intracellular degradation. Furthermore, these three variants caused significantly higher proliferation, migration, and invasion of colon cancer cells than the wild type. Our findings suggest that missense variants of *SLC22A18* can potentially serve as biomarkers or prognostic tools that enable clinicians to predict colorectal cancer progression.

## Introduction

Colorectal cancer ranks as the second most common cancer in women and the third most common in men worldwide^1^. In the United States, it is the third most diagnosed cancer in both sexes, with the third-highest overall cancer mortality rate. Despite a slight decrease in the overall incidence rate, there is a persistent rise in colorectal cancer cases among individuals under 55 years of age. Notably, despite an overall decline in colorectal cancer mortality, there has been an annual increase ranging from 0.5 to 3% among those under 50 years of age. Within the United States, colorectal cancer is the second leading cause of cancer-related deaths in men under 50 years of age^2^.

Transporters belonging to the solute carrier family (SLC) play crucial roles in maintaining physiological functions within the digestive system. These SLC transporters actively regulate nutrient absorption and facilitate the transportation of nutrients among cellular organelles^3^. Numerous studies have highlighted associations between the underexpression or overexpression of SLC transporters and the occurrence or progression of various digestive system cancers^4^. Notably, the overexpression of *SLC7A5*, *SLC29A2*, or *SLC38A1* is significantly associated with poor survival in patients with hepatocellular carcinoma^5,6^.

SLC22A18, which belongs to the *SLC22* gene family, is an orphan transporter with an unknown endogenous substrate, though it may be involved in transporting chloroquine and quinidine^7^. *SLC22A18* expression is methylation-dependent, and the transcription factor Sp1 transactivates the *SLC22A18* promoter^8,9^.

*SLC22A18* reportedly acts as a suppressor in various cancers, including prostate cancer, Wilms’ tumor, glioma, breast cancer, and colorectal cancer^10–15^. Particularly, Jung et al.^15^ revealed that *SLC22A18* is downregulated in colorectal cancer tissues, exerting suppressive effects by inhibiting colony formation and inducing G2/M arrest. They further reported a significant correlation between low *SLC22A18* expression and a poor long-term prognosis in patients with colorectal cancer. In contrast, *SLC22A18* is overexpressed in pancreatic cancer^16^.

Although many case–control studies aimed at gaining a better understanding of *SLC22A18* have been conducted, relatively few molecular and functional studies of *SLC22A18* genetic variants have been reported. In this study, we investigated the effects of four missense *SLC22A18* variants on transporter expression and the progression of colon cancer cells using various in vitro assay systems.

## Results

### Frequencies of *SLC22A18* missense variants

We examined common missense variants of *SLC22A18* using data from the Database of Single Nucleotide Polymorphisms (dbSNP) of the National Center for Biotechnology Information (NCBI) (https://www.ncbi.nlm.nih.gov/snp/) and identified four variants, namely p.Ala6Thr (rs1048046), p.Arg12Gln (rs1048047), p.Arg86His (rs78838117), and p.Trp324Cys (rs1129782). Subsequently, the frequencies of these variants in four different ethnic groups—661 Africans, 347 North Americans, 504 East Asians, and 503 Europeans—were obtained using frequency data from the 1000 Genomes Project (phase 3; https://www.ensembl.org/; Table 1). The wild type *SLC22A18* mRNA sequence was based on the reference *SLC22A18* mRNA sequence (GenBank accession number NM_183233.3).

**Table 1:** Frequencies of *SLC22A18* genetic variations in four different ethnic groups.

rs Number	Variant	Amino acid substitution	Minor allele	Minor allele frequency			
				East Asian	African	American	European
rs1048046	c.16G > A	p.Ala6Thr	A	0.005	0.268	0.095	0.193
rs1048047	c.35G > A	p.Arg12Gln	G	0.242	0.548	0.409	0.399
rs78838117	c.257G > A	p.Arg86His	A	0.061	–	0.003	–
rs1129782	c.972G > C	p.Trp324Cys	C	0.123	0.008	0.134	0.202

The data shown were obtained from the 1000 Genomes Project (phase 3) for 661 Africans, 347 Americans, 504 East Asians, and 503 Europeans.

### Effects of *SLC22A18* variations on its expression

**Figure 1:**
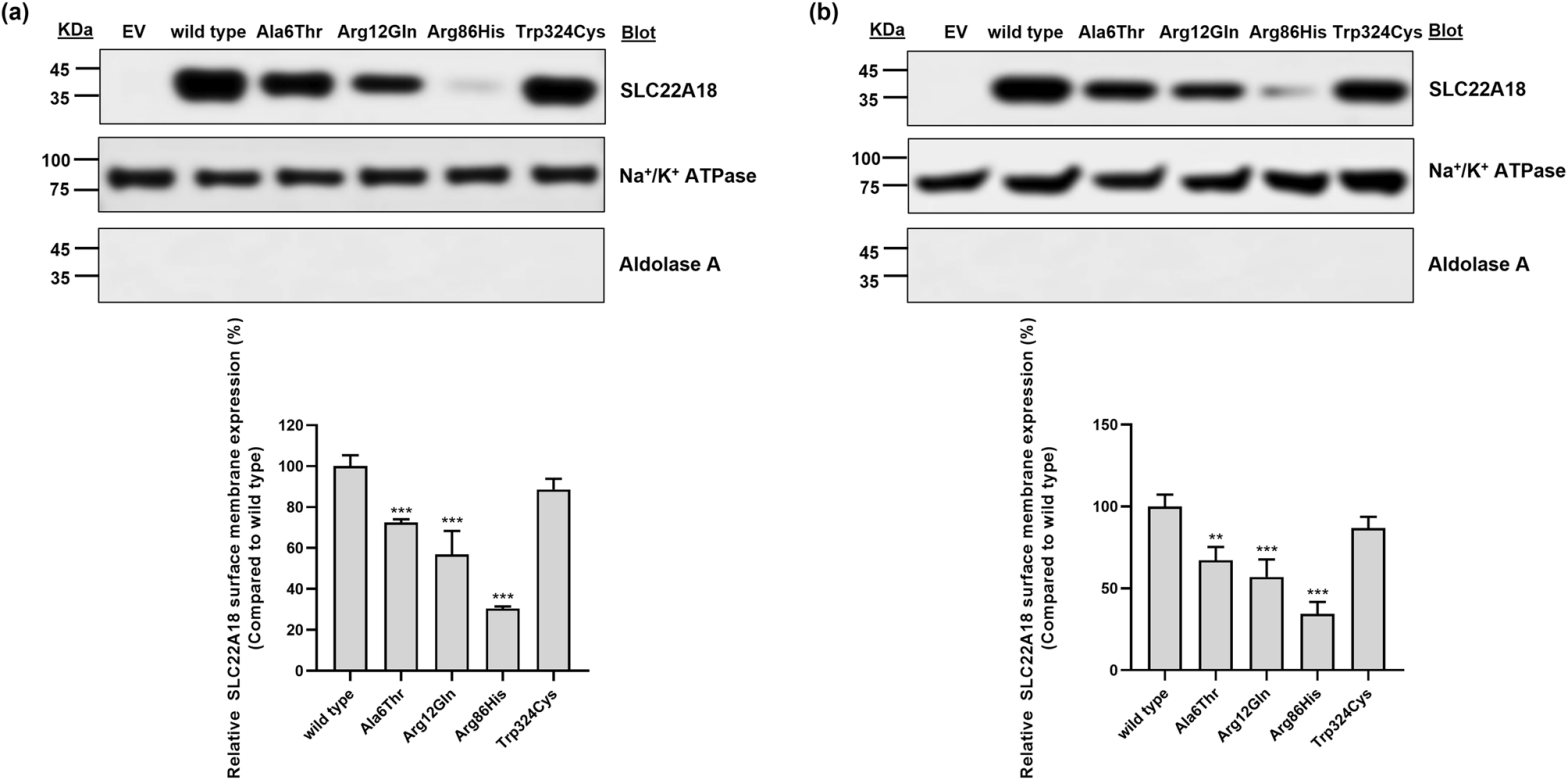
Effect of *SLC22A18* variants on SLC22A18 expression. A surface biotinylation assay was conducted using HCT-116 (**a**) or SW620 (**b**) cells stably expressing wild type *SLC22A18* or the its variant. Cropped images are displayed in this figure, and full-length images are presented in Supplementary Fig. 1. The data are presented as the mean ± standard deviation (SD) of three independent experiments analyzed using one-way analysis of variance followed by Dunnett’s two-tailed test; ^**^p < 0.01, ^***^p < 0.001 versus wild type. EV, empty vector.

We constructed vectors encoding wild type *SLC22A18* or four variants and generated separate stable cell lines expressing each gene. Subsequently, we investigated the expression levels of the wild type SLC22A18 protein and each variant via surface biotinylation assays. Three of the four missense variants—p.Ala6Thr, p.Arg12Gln, and p.Arg86His—showed significantly decreased SLC22A18 expression, with the expression levels of the p.Ala6Thr, p.Arg12Gln, and p.Arg86His variants being 27.6, 43.0, and 69.5% lower in HCT-116 cells, respectively, than that of the wild type (Fig. 1a). Significant expression decreases (32.8, 43.1, and 65.5%, respectively) were also observed in SW620 cells (Fig. 1b). The expression of p.Trp324Cys was comparable with that of the wild type in both types of cells (Fig. 1). Additionally, we examined the effect of each variant on *SLC22A18* mRNA expression using quantitative real-time polymerase chain reaction (qRT-PCR) analysis. We observed that the mRNA expression levels of the variants were comparable with that of the wild type (Supplementary Fig. 3). These results indicate that the decreased SLC22A18 expression of the three variants has resulted from post-transcriptional events.

**Figure 2:**
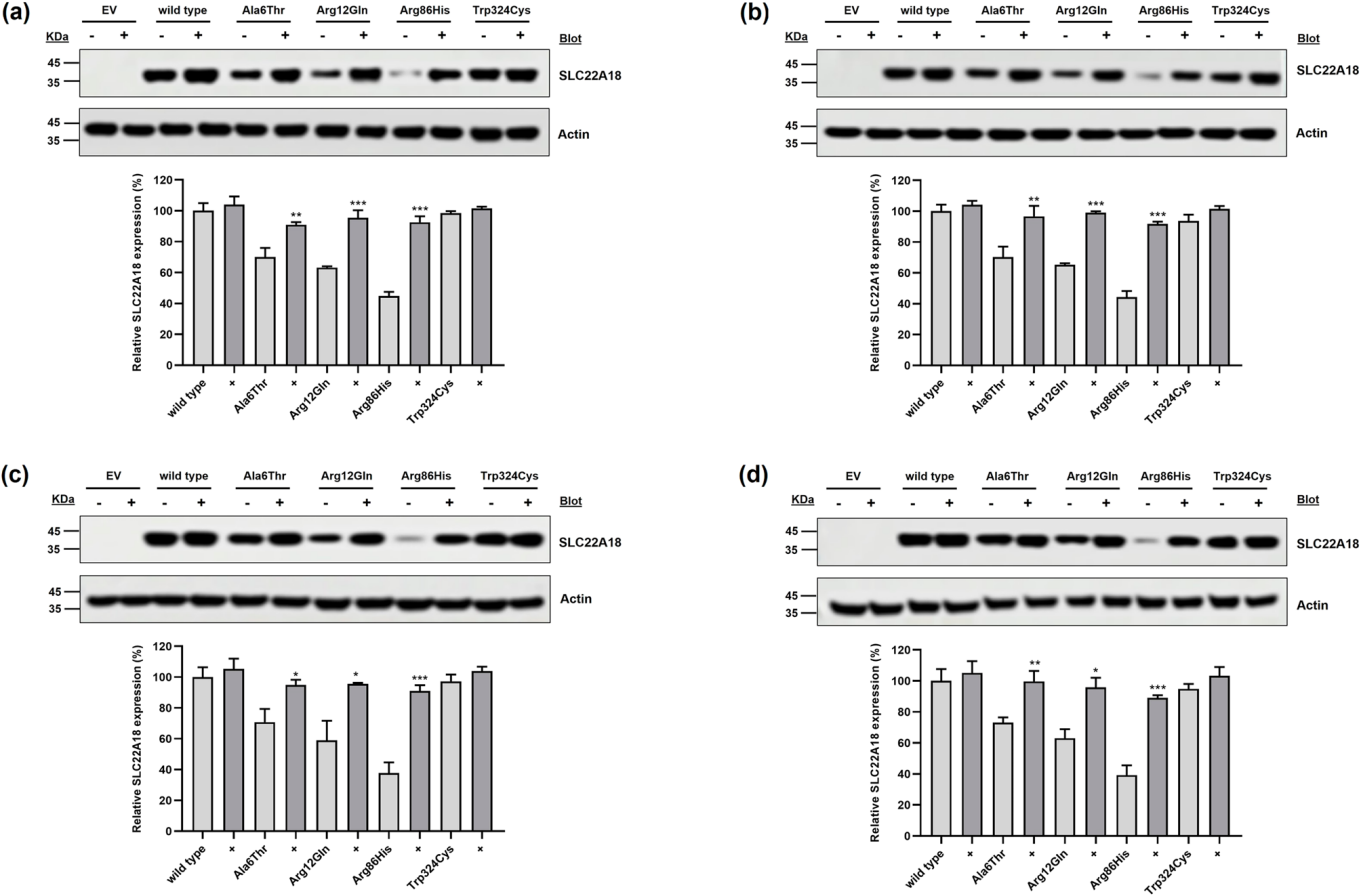
Effect of MG132 or bafilomycin A_1_ on SLC22A18 expression. Immunoblotting was performed following treatment with MG132 (**a, c**) or bafilomycin A_1_ (**b, d**) using stable HCT-116 (**a, b**) or SW620 (**c, d**) cells. Images were cropped, and full-length blots are presented in Supplementary Fig. 2. Data are presented as the mean ± SD of three independent experiments as determined using Student’s *t*-test; ^*^p < 0.05, ^**^p < 0.01, ^***^p < 0.001 versus native SLC22A18 expression without MG132 or bafilomycin A_1_ treatment. EV, empty vector.

**Figure 3:**
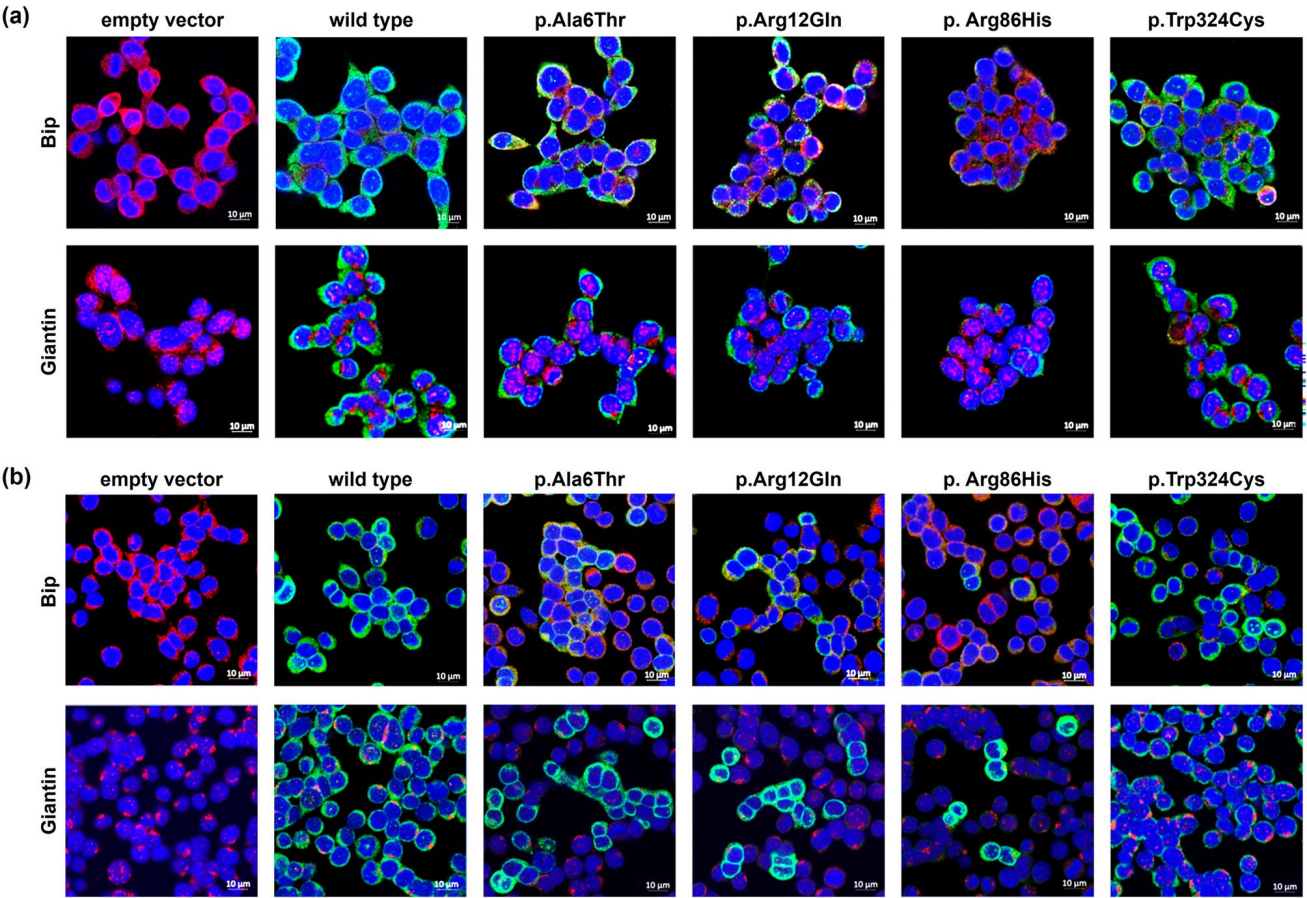
Effects of *SLC22A18* variants on subcellular localization. Stable HCT-116 (**a**) or SW620 (**b**) cells were immunostained using primary antibodies against FLAG M2, an endoplasmic reticulum marker (BiP), or a Golgi marker (giantin). Scale bars, 10 µm.

To elucidate the mechanisms underlying the reduced membrane expression of *SLC22A18* variants, we conducted immunoblotting following pre-treatment with MG132 (a proteasomal proteolysis inhibitor) and bafilomycin A_1_ (a lysosomal degradation inhibitor). Post-MG132 treatment, the expression levels of the p.Ala6Thr, p.Arg12Gln, and p.Arg86His variants in HCT-116 cells recovered to 91.0%, 95.6%, and 92.6%, respectively, compared with that of the wild type (Fig. 2a). Additionally, the expression of these variants in HCT-116 cells recovered to 96.6%, 99.1%, and 91.8%, respectively, of that of the wild type after bafilomycin A_1_ treatment (Fig. 2b). In SW620 cells, expression of the p.Ala6Thr, p.Arg12Gln, and p.Arg86His variants significantly recovered to 94.9%, 95.6%, and 91.0%, respectively, following MG132 treatment (Fig. 2c) and to 99.6%, 95.9%, and 89.1%, respectively, after bafilomycin A_1_ treatment (Fig. 2d). Our findings suggest that p.Ala6Thr, p.Arg12Gln, and p.Arg86His are susceptible to intracellular degradation, and the diminished expression of these *SLC22A18* variants may be attributed to proteasomal or lysosomal degradation. Additionally, we performed immunofluorescence staining to confirm the expression of the *SLC22A18* variants on the plasma membrane. We observed that the SLC22A18 expression of the three variants, p.Ala6Thr, p.Arg12Gln, and p.Arg86His, was decreased on the plasma membranes of both HCT-116 and SW620 cells (Fig. 3a,b, respectively). Expression of the p.Arg86His variant was particularly decreased. Larger factions of the p.Ala6Thr and p.Arg12Gln variants were present in the endoplasmic reticulum compared with that of the wild type.

### Effects of *SLC22A18* variants on the proliferation, migration, and invasion of colon cancer cells

**Figure 4:**
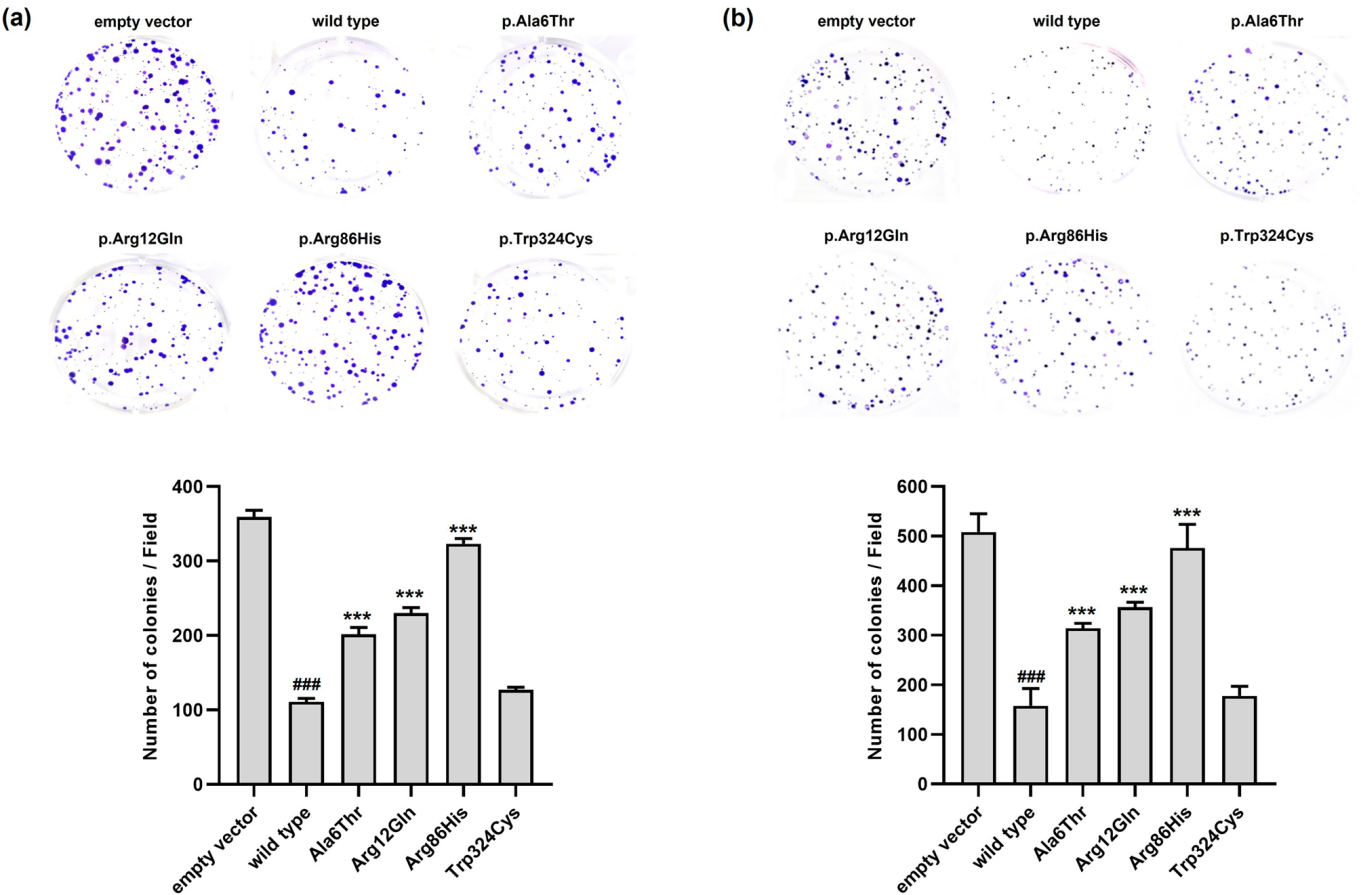
Effects of *SLC22A18* variants on the proliferation of colon cancer cells. Stable HCT-116 (**a**) or SW620 (**b**) cells were cultured for 10 days, the colonies were stained with crystal violet solution, and the colonies were counted. Data are presented as the mean ± SD of three independent experiments as determined via one-way analysis of variance followed by Dunnett’s two-tailed test; ^###^*p* < 0.001 versus empty vector; ^***^p < 0.001 versus wild type.

**Figure 5:**
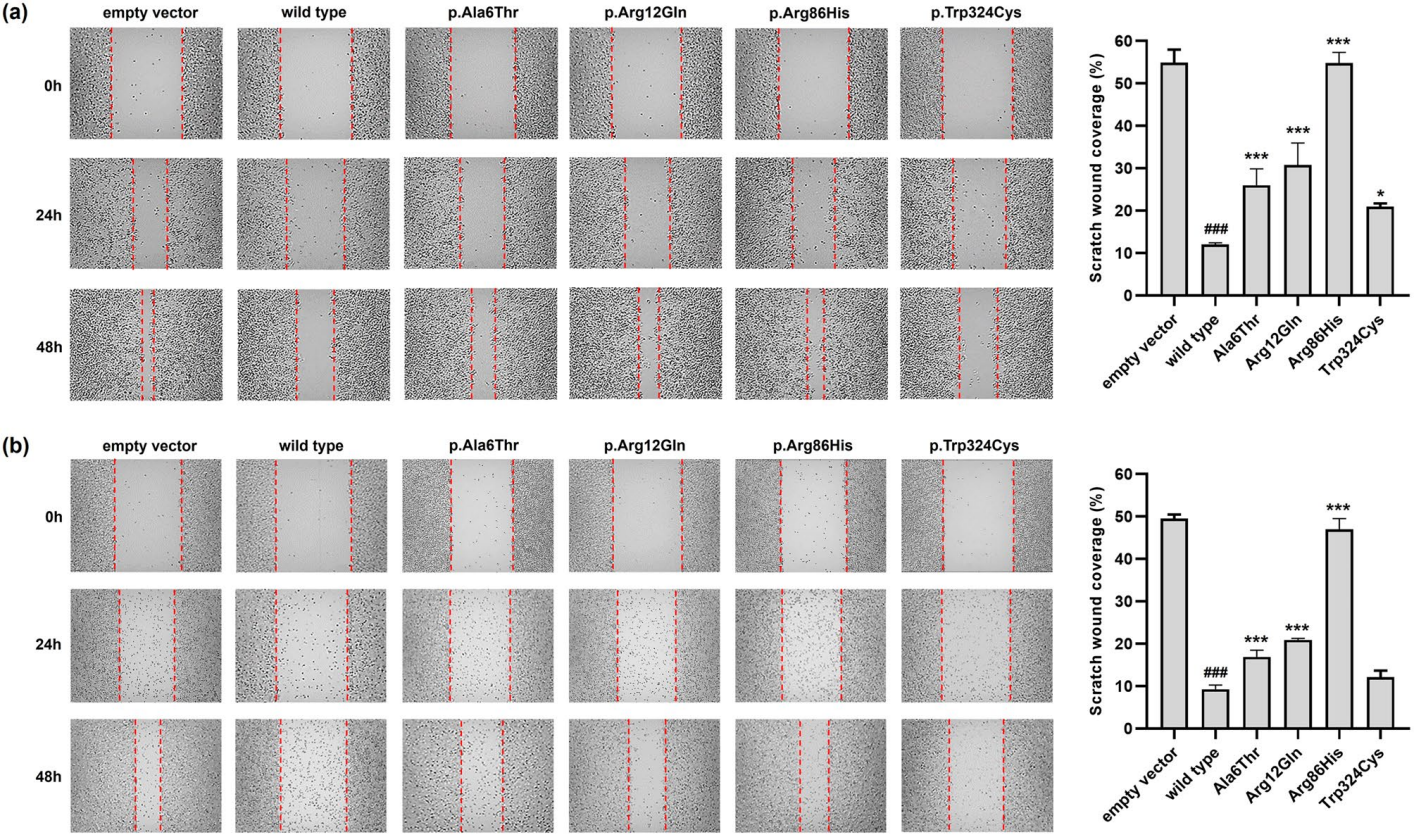
Effects of *SLC22A18* variants on the migration of colon cancer cells. Stable HCT-116 (**a**) or SW620 (**b**) cells were cultured to 80–90% confluency, and the monolayers were gently scratched with a scratcher. Images were acquired after 0, 24, and 48 h. Data after 48 h are presented as the mean ± SD of three independent experiments, as determined via one-way analysis of variance followed by Dunnett’s two-tailed test; ^###^*p* < 0.001 versus empty vector; ^*^p < 0.05, ^***^p < 0.001 versus wild type.

**Figure 6:**
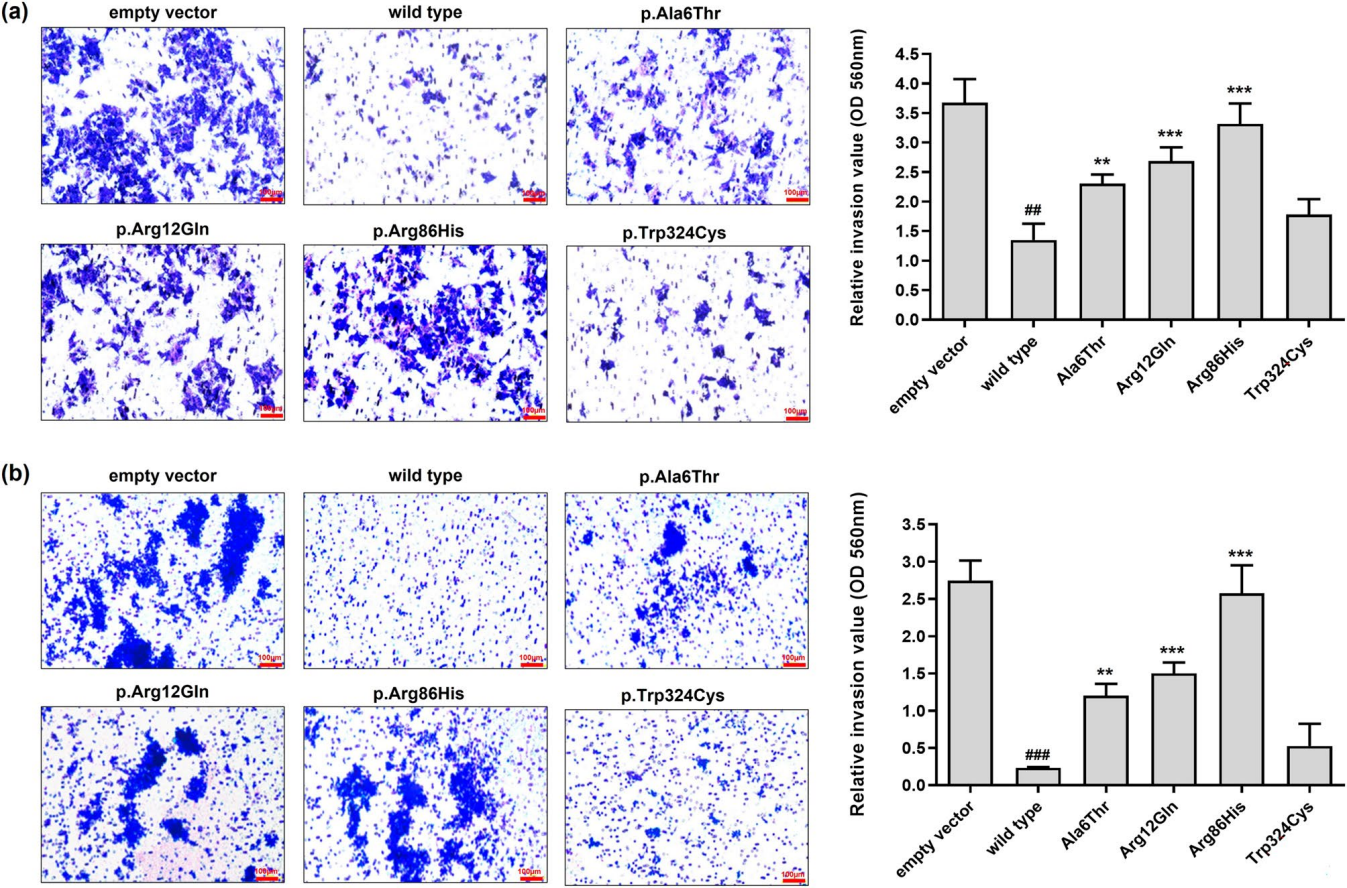
Effects of *SLC22A18* variants on the invasion of colon cancer cells. After incubating stable HCT-116 (**a**) or SW620 (**b**) cells on inserts in invasion chambers for 48 h, they were stained with the staining solution for 20 min. Subsequently, the number of cells was counted. Data are presented as the mean ± SD of three independent experiments, as determined via one-way analysis of variance followed by Dunnett’s two-tailed test; ^##^*p* < 0.01, ^###^*p* < 0.001 versus empty vector; ^**^p < 0.01, ^***^p < 0.001 versus wild type.

We conducted colony formation, wound healing, and invasion assays to investigate whether the *SLC22A18* variants might affect the proliferation, migration, and invasion of colon cancer cells. Colony formation was dramatically lower in HCT-116 and SW620 cells expressing wild type *SLC22A18* than in those expressing the empty vector (Fig. 4a,b, respectively). These results are consistent with previous findings showing that *SLC22A18* can suppress colorectal cancer^15^. The p.Ala6Thr, p.Arg12Gln, and p.Arg86His showed 1.8-, 2.1-, and 2.9-fold higher colony formation, respectively, than that of the wild type in HCT-116 cells (Fig. 4a). In addition, the variants in SW620 cells increased colony formation by 2.0-, 2.3-, and 3.0-fold, respectively, relative to the wild type (Fig. 4b). Our wound healing assays revealed that post-scratch wound coverage was lower in HCT-116 cells expressing the wild type protein (12.0%) compared with that of the empty vector (54.9%) (Fig. 5a). Similar results were observed in experiments with SW620 cells (9.3% and 49.5% coverage, respectively; Fig. 5b). In HCT-116 cells, the p.Ala6Thr, p.Arg12Gln, and p.Arg86His variants significantly increased wound coverage by 26.0%, 30.8%, and 54.8%, respectively (Fig. 5a). In SW620 cells, the respective wound coverages increased significantly to 16.9%, 20.9%, and 47.0%, respectively (Fig. 5b). Finally, we observed that invasion was markedly lower with HCT-116 or SW620 cells expressing the wild type protein than the empty vector (Fig. 6). The p.Ala6Thr, p.Arg12Gln, and p.Arg86His variants significantly increased HCT-116 cell invasion by 1.7-, 2.0-, and 2.5-fold, respectively, compared with that of wild type protein (Fig. 6a). In SW620 cells, the variants increased cell invasion by 5.1-, 6.4-, and 10.9-fold, respectively, compared with that of the wild type (Fig. 6b).

### Effects of the *SLC22A18* variants on oxaliplatin sensitivity

**Figure 7:**
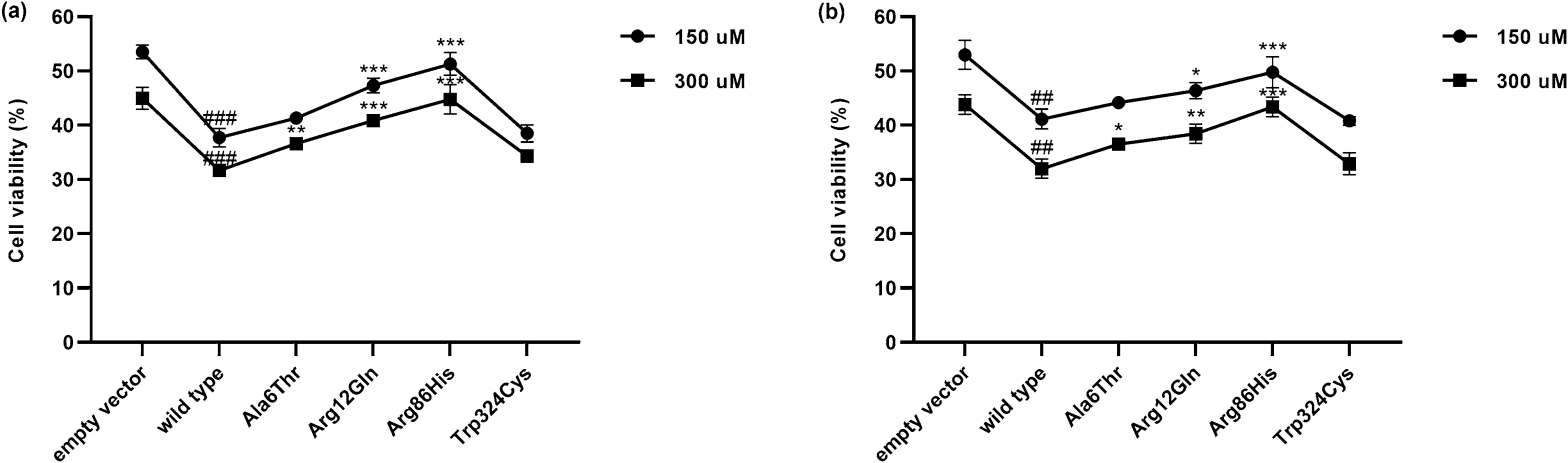
Effects of *SLC22A18* variants on the sensitivity to oxaliplatin. Various doses (0, 150, or 300 µM) of oxaliplatin were added to stable HCT-116 (**a**) or SW620 (**b**) cells. After 24 h, cell viability assays were performed. Data are presented as the mean ± SD of three independent experiments, as determined via one-way analysis of variance followed by Dunnett’s two-tailed test; ^##^*p* < 0.01, ^###^*p* < 0.001 versus empty vector; ^*^p < 0.05, ^**^p < 0.01, ^***^p < 0.001 versus wild type.

A previous report showed that low *SLC22A18* expression correlated significantly with oxaliplatin resistance^17^. In this study, we examined the effects of the *SLC22A18* variants on oxaliplatin sensitivity by performing cell viability assays after treatment of various doses (0, 150, and 300 µM) of oxaliplatin. Then, the cell viabilities observed with 150 or 300 µM oxaliplatin were calculated and statistically analyzed relative to those observed in the absence of oxaliplatin. The viability of cells expressing the wild type protein was significantly lower than that of empty vector. Two variants (p.Arg12Gln and p.Arg86His) were associated with significantly increased cell viabilities than the wild type. The viability of cells expressing the p.Ala6Thr variant tended to be higher than that of cells expressing the wild type protein, and this difference was statistically significant after treatment with 300 µM oxaliplatin, whereas p.Trp324Cys and wild type showed similar viabilities (Fig. 7).

## Discussion

Various SLC transporters are expressed in the digestive system, wherein they help maintain normal physiological functions by transporting various molecules, such as ions, metals, glucose, amines, and amino acids^18–20^. Many previous reports showed significant associations between SLC transporters and the occurrence and prognosis of digestive system cancers^4^. In particular, many SLC transporters, such as SLC5A8, SLC11A2, SLC12A2, SLC12A5, SLC22A18, SLC25A22, SLC26A2, SLC26A3, and SLC40A1, have been associated with colorectal cancer^15,21–28^. Among them, *SLC5A8*, *SLC22A18*, *SLC26A3*, and *SLC40A1* were either downregulated in colorectal cancer or found to protect against colorectal cancer by acting as tumor suppressors^15,21,22,27–29^. The remaining transporters are known for being overexpressed in colorectal cancer or being associated with the progression and poor prognosis of colorectal cancer^23,24,26,30,31^.

Although many investigators have commented on the association between SLC transporters and digestive system cancers, few have revealed the roles played by different transporters in the occurrence or progression of cancer. The tumor-suppressor role played by *SLC5A8* in colorectal cancer is one example of a known mechanism. SLC5A8 transports bacterial products (butyrate and propionate) and a ubiquitous metabolite (pyruvate) within the body. Evidently, the SLC5A8 transporter ultimately helps suppress colorectal cancer by transporting these substances, which inhibit histone deacetylase in cancer cells^29,32–36^. In contrast to *SLC5A8*, *SLC25A22* reportedly plays a tumor-promoter role in colorectal cancer. Wong et al.^25^ revealed that *SLC25A22* was essential for the biosynthesis of aspartate, which metabolizes into asparagine, an amino acid involved in the migration of colorectal cancer cells via the mitogen-activated protein kinase (MEK)/extracellular-signal-regulated kinase (ERK) pathway. Another metabolite of aspartate, oxaloacetate (which promotes intracellular glycolysis and reduces oxidative stress), is also involved in the proliferation and survival of colorectal cancer cells. In addition, *SLC25A22* promoted the development and metastasis of colon cancer in mice, and colorectal cancer patients overexpressing *SLC25A22* have a shorter survival time than patients with low *SLC25A22* expression.

Several previous reports showed that *SLC22A18* may suppress various cancers, including prostatic cancer, Wilms’ tumor, glioma, breast cancer, and colorectal cancer^10–15^. A previous report showed that *SLC22A18* expression was significantly lower in patients with colorectal cancer and that low *SCL22A18* expression correlated with lower long-term survival in these patients^15^. In this report, the authors also revealed that *SLC22A18* inhibited colony formation by colon cancer cells and induced cell-cycle arrest in the G2/M phase. Furthermore, *SL22A18* inhibited the growth of HCT-116 xenograft tumors in a mouse model. Recently, another report showed that low *SLC22A18* expression in patients with colorectal cancer correlated significantly with oxaliplatin resistance and that this resistance could be overcome by inhibiting the ERK pathway^17^.

In this study, we examined the expression levels of four missense variants of *SLC22A18* and their effects on the progression of colon cancer cells. We observed that three variants—p.Ala6Thr, p.Arg12Gln, and p.Arg86His—had significantly lower cell expression than the wild type, and these variants increased the proliferation, migration, and invasion of colon cancer cells. Our results are consistent with those of a previous report indicating that *SLC22A18* may suppress colorectal cancer^15^. Furthermore, we observed that p.Ala6Thr, p.Arg12Gln, and p.Arg86His significantly affected the sensitivity of cells to oxaliplatin. Therefore, disease progression and responses to oxaliplatin in patients with colorectal cancer may vary depending on the presence or absence of *SLC22A18* variants. To confirm this possibility, additional analysis of clinical patient data based on genetic variations will be necessary.

Screening for colorectal cancer via colonoscopy has reduced the incidence and mortality rates of colorectal cancer by approximately 40–60%, respectively^37–39^. Recently published statistical data indicate that although the overall annual incidence of colorectal cancer appears to be slightly decreasing, it has been increasing by 1–2% per year in people under 55 years of age. Moreover, even though the overall mortality rate of colorectal cancer is decreasing every year, the mortality rates for people under 50 years of age have been increasing by 0.5 to 3% each year. Additionally, colorectal cancer is often diagnosed at more advanced stages than in the past^2^. Thus, to reduce the incidence and mortality rates of colorectal cancer, especially in people under 50–55 years of age, continual efforts should be undertaken to develop biomarkers that can help diagnose colorectal cancer more accurately and quickly, as well as more effective treatment methods. Numerous studies have been conducted to search for useful screening biomarkers for colorectal cancer, which have identified many molecular markers. For example, various types of microRNAs and long non-coding RNAs in blood or stool samples have been significantly correlated with colon cancer, and some tissue biomarkers (including caudal type homeobox 2, special AT-rich sequence-binding protein 2, and glycoprotein A33) have been identified. Recently, the methylated gene, *septin* 9 (mSEPT9) was approved by the Food and Drug Administration as a new biomarker for colorectal cancer^40^.

Several SLC transporters are either overexpressed or underexpressed in colorectal cancer, and differences in some of their expression have been associated with the prognoses of patients with colorectal cancer^15,21–31^. Therefore, genetic variants that significantly affect the expression or function of SLC transporters can be used as biomarkers for diagnosing or predicting the progression of colorectal cancer. In addition, as exemplified by the association between *SLC22A18* and oxaliplatin resistance, genetic variants may be used to predict drug resistance or select drugs during chemotherapy in patients with colorectal cancer^17^.

To the best of our knowledge, this study is the first to investigate the effects of missense variants of *SLC22A1*8 on transporter expression, colon cancer cell progression, and oxaliplatin sensitivity. Further studies are needed to evaluate the clinical utility of the variants that showed significant effects in vitro.

## Methods

### Genetic analysis of *SLC22A18* missense variants

Data from the dbSNP of NCBI were used to identify common missense variants of *SLC22A18*. Next, the frequencies of each variant in four different ethnic groups were obtained from the 1,000 Genomes Project (phase 3). The numbers of people represented in each group, the population descriptions, and the population codes are as follows: 661 Africans (African Caribbean in Barbados [ACB], African Ancestry in Southwest US [ASW], Esan in Nigeria [ESN], Gambian in Western Division, The Gambia–Mandinka [GWD], Luhya in Webuye, Kenya [LWK], Mende in Sierra Leone [MSL], and Yoruba in Ibadan, Nigeria [YRI]); 347 Americans (Colombian in Medellin, Colombia [CLM], Mexican Ancestry in Los Angeles, California [MXL], Peruvian in Lima, Peru [PEL], and Puerto Rican in Puerto Rico [PUR]]; 504 east Asians (Chinese Dai in Xishuangbanna, China [CDX], Han Chinese in Beijing, China [CHB], Han Chinese South [CHS], Japanese in Tokyo, Japan [JPT], and Kinh in Ho Chi Minh City, Vietnam [KHV]); and 503 Europeans [Utah residents with Northern and Western European ancestry [CEU], Finnish in Finland [FIN], British in England and Scotland [GBR], Iberian populations in Spain [IBS], and Toscani in Italy [TSI]).

### Plasmid construction

Complementary DNA (cDNA) encoding wild type *SLC22A18* (SC319659, OriGene) was subcloned into the p3XFLAG-CMV vector. The variant-bearing plasmids were generated using a QuikChange® II Site-Directed Mutagenesis Kit (Agilent Technologies). The sequences of all primers used for plasmid construction are listed in Supplementary Table 1. The DNA sequences of all plasmids were confirmed via direct sequencing.

### Selection of stable cell lines

To generate cell lines stably expressing wild type *SLC22A18* or its variants, plasmids encoding wild type *SLC22A18* or its variants were separately transfected into HCT-116 (human colorectal carcinoma) or SW620 (human Caucasian colon adenocarcinoma) cells (Korean Cell Line Bank, Korean Cell Line Research Foundation) using the Lipofectamine® LTX and Plus™ reagents (Invitrogen Corporation). The cells were then cultured for 5 days in medium containing 8 mg/ml of neomycin antibiotic G418 (InvivoGen). Individual colonies were isolated and cultured with decreasing concentrations of G418.

### Surface biotinylation assay

Biotinylation assays were performed using a Cell Surface Protein Isolation Kit (Thermo Fisher Scientific) according to the manufacturer’s protocol. A rabbit polyclonal anti-Na^+^/K^+^ ATPase α-1 antibody (Merck) was used as an internal standard. The signal was acquired using ImageQuant LAS 4000 mini (GE Healthcare), and the intensity of each band was measured using ImageJ software (National Institute of Health).

### Immunoblotting

Immunoblotting was performed using a mouse anti-FLAG M2 primary antibody (Sigma-Aldrich) or a rabbit anti-β-actin antibody (Merck). Cells were treated with 10 µM MG132 (Sigma-Aldrich) or 10 nM bafilomycin A_1_ (MedChemExpress) at 24 h post-transfection to examine their effects on expression of the *SLC22A18* variants. Signals were obtained using an ImageQuant LAS 4000 mini, and the intensity of each band was measured using ImageJ software.

### qRT-PCR analysis

Total RNA was extracted from cells with TRIzol (Invitrogen Corporation) according to the manufacturer’s protocol. Subsequently, reverse transcription was performed using 1 µg of RNA and the iScript cDNA Synthesis Kit (Bio-Rad). mRNA expression of wild type *SLC22A18* or each variant was analyzed via qRT-PCR. qRT-PCR was conducted with SYBR Green Real-time PCR Master Mix (Toyobo) and QuantStudio™ 3 Real-Time PCR machine (Thermo Fisher Scientific). mRNA expression levels were normalized to that of *GAPDH* mRNA. The sequences of the primers used for the qRT-PCR step (Cosmogenetech) are shown in Supplementary Table 1.

### Immunofluorescence

HCT-116 or SW620 stable cells were grown in 4-well chamber slides (Thermo Fisher Scientific) and allowed to attach for 1 day. Cells were fixed and permeabilized with 100% methanol (prechilled at − 20 °C) at room temperature for 5 min and blocked with 1% bovine serum albumin for 1 h. Then, the cells were incubated with anti-FLAG M2, anti-BiP (Abcam), or anti-giantin (Abcam) antibodies. After washing cells with phosphate-buffered saline (PBS), they were incubated with secondary Alexa Fluor® 488-conjugated rabbit anti-mouse IgG or Alexa Fluor® 594-conjugated goat anti-rabbit IgG (Thermo Fisher Scientific) antibodies. Nucleic acids were stained with 4′,6-diamidino-2-phenylindole (Vector Laboratories). Images were acquired using a confocal laser-scanning microscope and analyzed using an LSM image examiner (Carl Zeiss).

### Colony formation assay

Stable HCT-116 or SW620 cells were seeded into 6-well plates (500 cells/well) and cultured for 10 days. The cells were then washed with PBS and fixed with methanol and acetic acid. The colonies were stained with crystal violet solution (Sigma-Aldrich) for 30 min at room temperature, and then the colonies were counted.

### Wound healing assay

Stable HCT-116 or SW620 cells were cultured to 80–90% confluence, after which their monolayers were gently scratched with a scratcher (SPL Lifesciences), and the floating cells were removed. Images were acquired using an IN300T-FL microscope (AmScope) after 0, 24, and 48 h.

### Cell invasion assay

Cell invasion was studied using a QCM ECMatrix Cell Invasion Assay, 24-Well (8 µm), Colorimetric Kit (EMD Millipore) according to the manufacturer’s protocol. Briefly, after incubating stable HCT-116 or SW620 cells on the inserts in invasion chambers for 48 h, they were stained with 500 µl of staining solution for 20 min. Finally, the number of cells was counted under an inverted microscope (TE-300, Nikon).

### Cell viability assay after oxaliplatin treatment

Stable HCT-116 or SW620 cells were grown in 96-well plates. Various doses (0, 150, or 300 µM) of oxaliplatin (Sigma-Aldrich) were added to the HCT-116 or SW620 cells growing in the 96-well plates. After 24 h, cell viabilities were measured using Cell Counting Kit-8 (Dojindo Laboratories).

### Statistical analyses

Statistical analyses were performed using GraphPad Prism 8.0 (GraphPad Software). We calculated *p* values to compare the results obtained before and after MG132 or bafilomycin A_1_ treatment using Student’s *t*-test. Other *p* values were calculated using one-way analysis of variance, followed by Dunnett’s two-tailed test. The threshold for statistical significance was set to *p* < 0.05.

### Supplementary Information

Supplementary Information.

## Data Availability

All data generated or analyzed during the current study are included in this article (and its supplementary information files).
