# Supplementary material for: The effect of genetic variants of SLC22A18 on proliferation, migration, and invasion of colon cancer cells
Source: Sci Rep. 2024 Feb 16;14:3925. doi: 10.1038/s41598-024-54658-w (PMC10873386; doi:10.1038/s41598-024-54658-w)
Supplement: Supplementary file 1 — Supplementary Information. [file 41598_2024_54658_MOESM1_ESM.pdf]

Supplementary Information For

The effect of genetic variants of *SLC22A18* on proliferation, migration, and invasion of colon cancer cells

Hyo Sook Song[1], Seung Yeon Ha[1], Jin-Young, Kim[1], Minsuk Kim[1], Ji Ha Choi[1,*]

[1]Department of Pharmacology, Inflammation-Cancer Microenvironment Research Center, College of Medicine, Ewha Womans University, Seoul, Republic of Korea

[*]Corresponding author: jihachoi@ewha.ac.kr

Correspondence to

Ji Ha Choi, M.D., Ph.D.

Department of Pharmacology, Inflammation-Cancer Microenvironment Research Center, College of Medicine, Ewha Womans University, 25 Magokdong-ro 2-gil, Gangseo-gu, Seoul 07804, Republic of Korea

Phone: +82-2-6986-6173; Fax: +82-2-6986-7017; E-mail: jihachoi@ewha.ac.kr

Supplementary Figure 1. Effect of *SLC22A18* variants on SLC22A18 expression. HCT-116 **(a)** or SW620 **(b)** cells were transfected with *SLC22A18* wild type or variant plasmids, and a surface biotinylation assay was conducted. Among figures, the figures named 'Repeat 1' were used in the main manuscript.

(a)

Repeat 1

Blot: SLC22A18 **Blot: Na^+/K^+ ATPase** **Blot: Aldolase A**

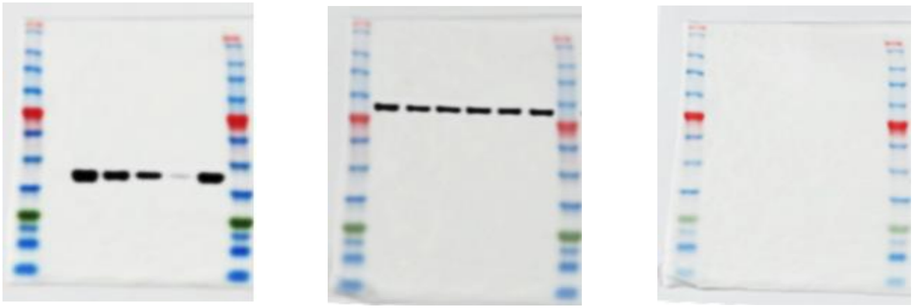

Repeat 2

Blot: SLC22A18 **Blot: Na^+/K^+ ATPase** **Blot: Aldolase A**

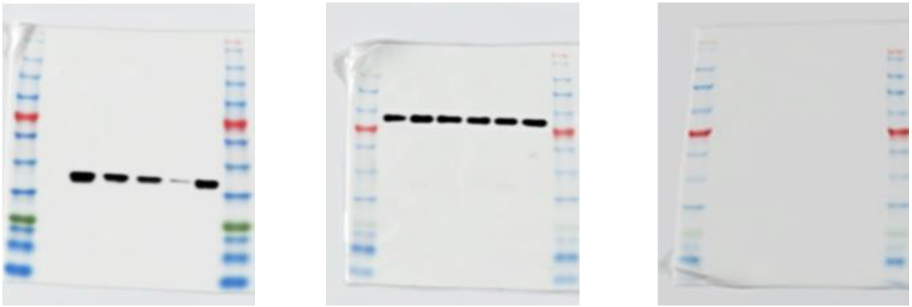

Repeat 3

Blot: SLC22A18 **Blot: Na^+/K^+ ATPase** **Blot: Aldolase A**

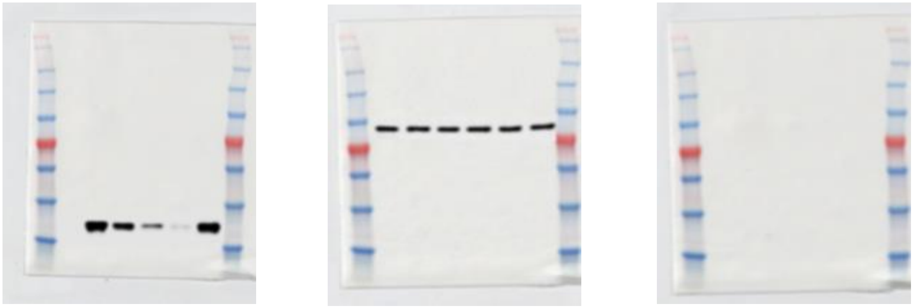

(b)

Repeat 1

Blot: SLC22A18

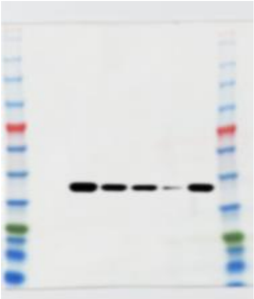

Blot: Na^{+}/K^{+} ATPase

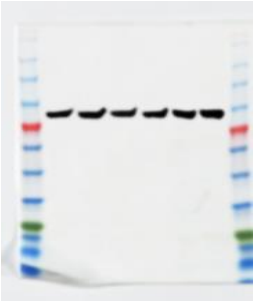

Blot: Aldolase A

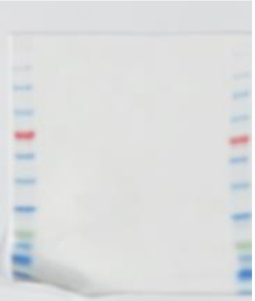

Repeat 2

Blot: SLC22A18

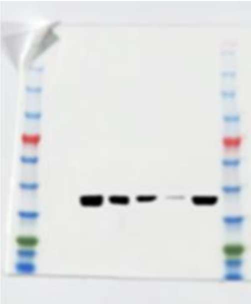

Blot: Na^{+}/K^{+} ATPase

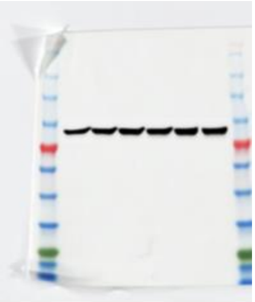

Blot: Aldolase A

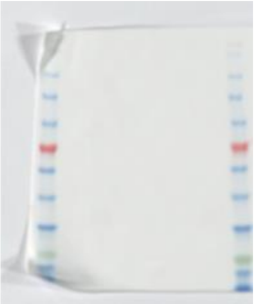

Repeat 3

Blot: SLC22A18

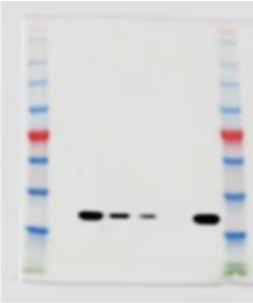

Blot: Na^{+}/K^{+} ATPase

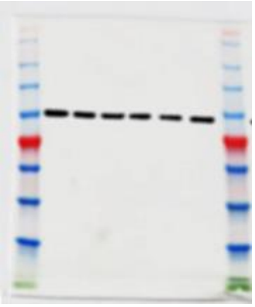

Blot: Aldolase A

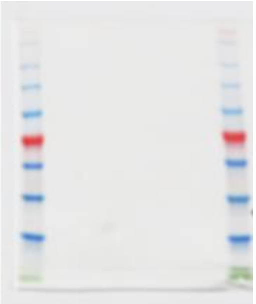

Supplementary Figure 2. Effect of MG132 or bafilomycin A_1 on SLC22A18 expression. SLC22A18 expression was examined after transfection with *SLC22A18* wild type or variant plasmids. Immunoblotting was performed after treatment with MG132 **(a, c)** or bafilomycin A_1 **(b, d)** in HCT-116 **(a, b)** or SW620 **(c, d)** cells. Among figures, the figures named 'Repeat 1' were used in the main manuscript.

(a)

Repeat 1

Blot: SLC22A18

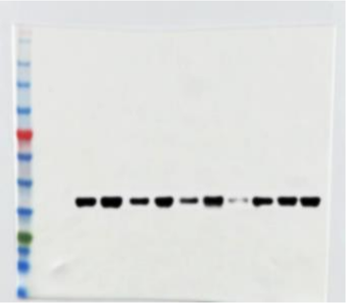

Blot: Actin

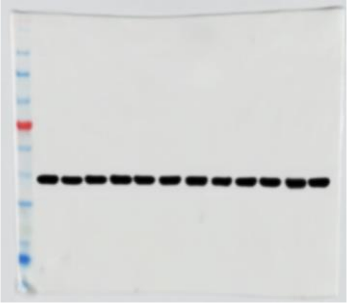

Repeat 2

Blot: SLC22A18

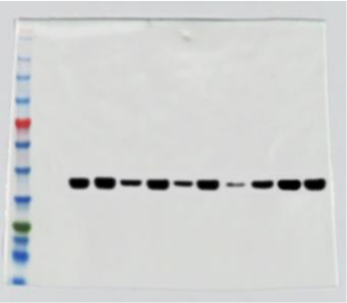

Blot: Actin

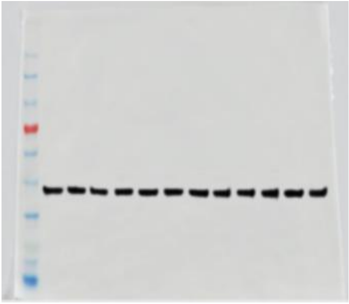

Repeat 3

Blot: SLC22A18

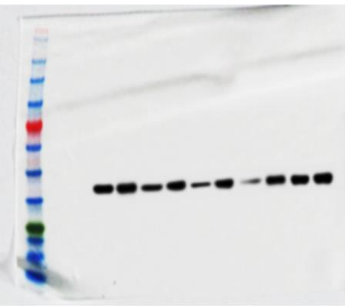

Blot: Actin

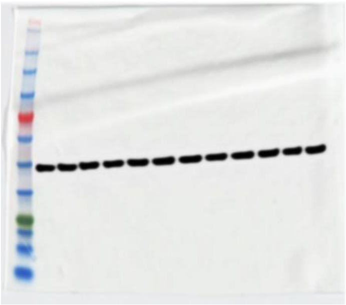

(b)

Repeat 1

Blot: SLC22A18

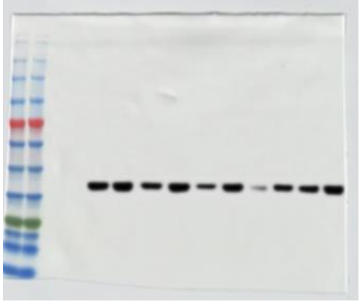

Blot: Actin

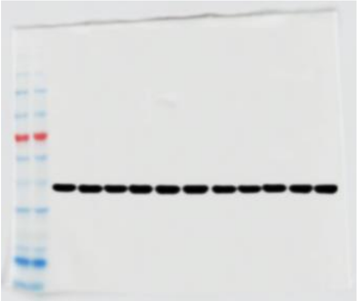

Repeat 2

Blot: SLC22A18

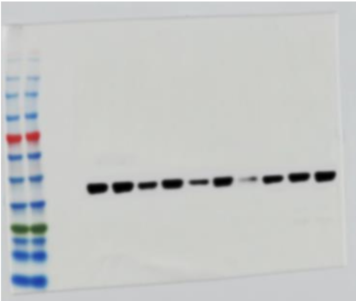

Blot: Actin

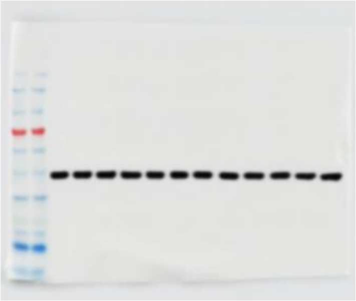

Repeat 3

Blot: SLC22A18

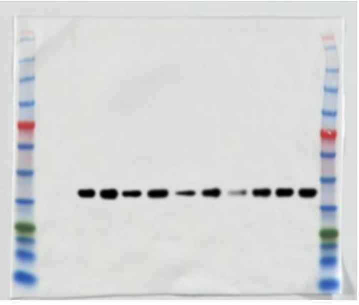

Blot: Actin

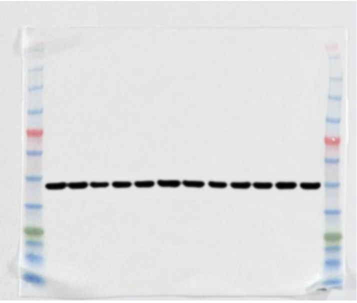

(c)

Repeat 1

Blot: SLC22A18

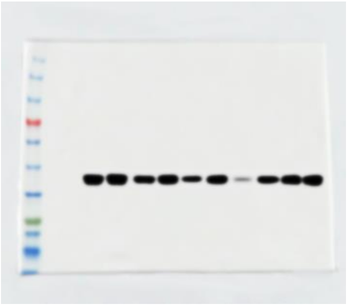

Blot: Actin

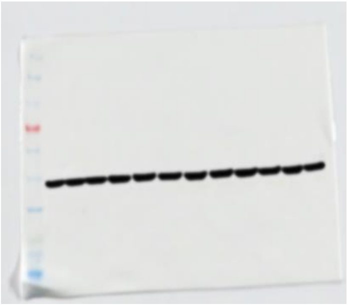

Repeat 2

Blot: SLC22A18

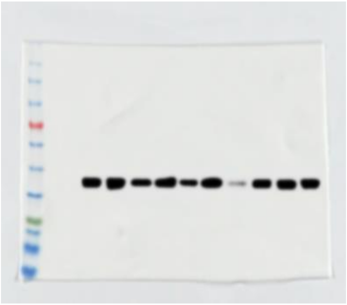

Blot: Actin

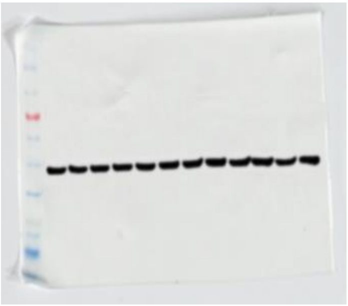

Repeat 3

Blot: SLC22A18

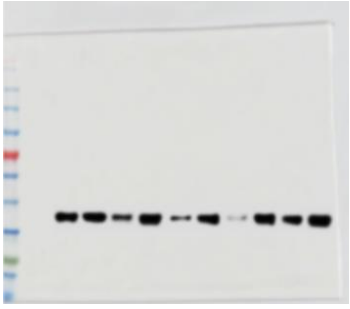

Blot: Actin

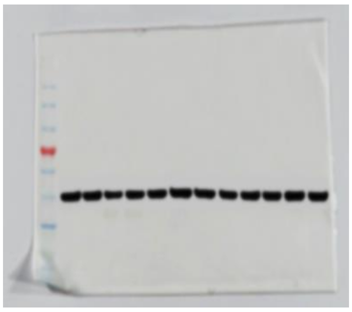

(d)

Repeat 1

Blot: SLC22A18

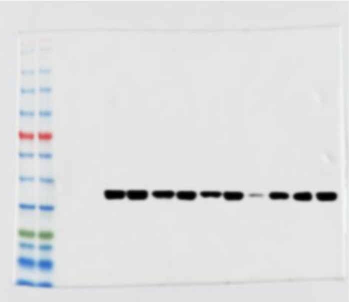

Blot: Actin

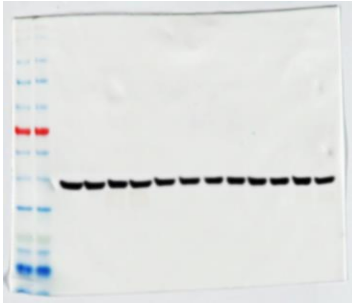

Repeat 2

Blot: SLC22A18

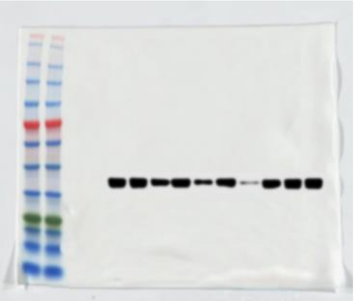

Blot: Actin

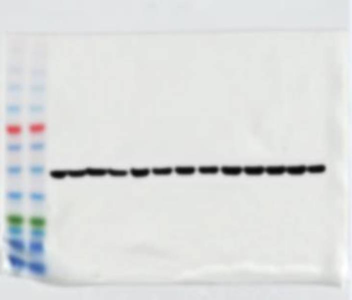

Repeat 3

Blot: SLC22A18

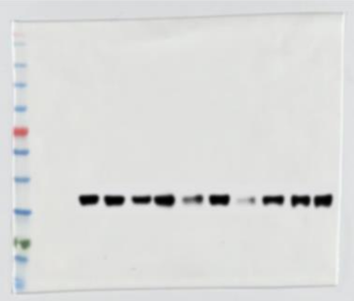

Blot: Actin

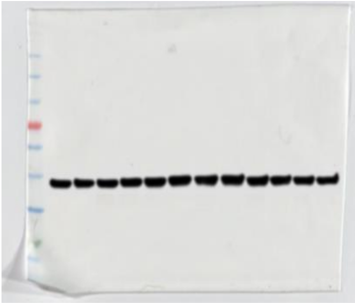

Supplementary Figure 3. Effects of *SLC22A18* variants on *SLC22A18* mRNA expression. qRT-PCR was performed to examine the mRNA expression levels of *SLC22A18* variants, using cDNA synthesized with reverse transcriptase and total RNA obtained from HCT-116 **(a)** or SW620 **(b)** cells. Data are presented as the mean ± SD of three independent experiments, as determined via one-way analysis of variance followed by Dunnett's two-tailed test.

(a)

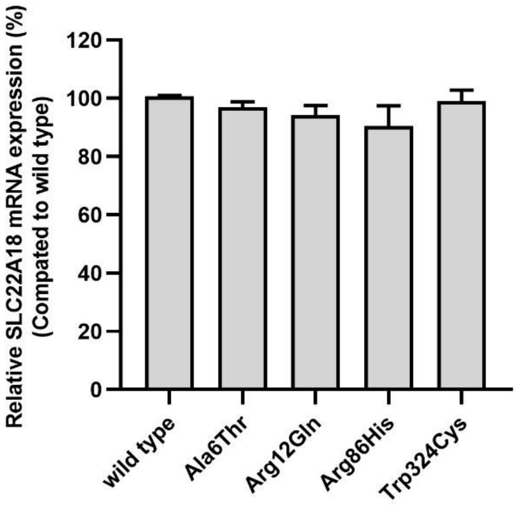

(b)

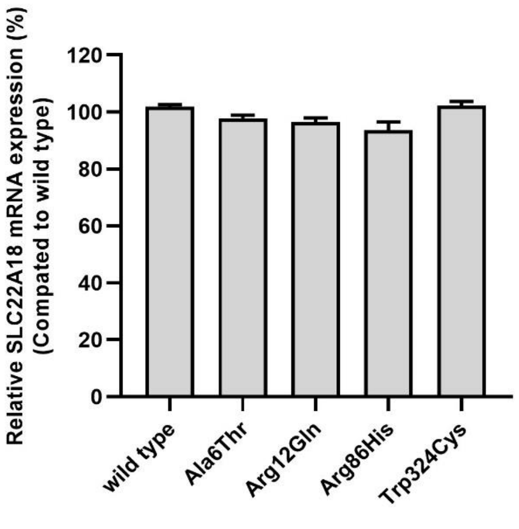

Supplementary Table 1. Primers used in the construction of *SLC22A18* plasmids or qRT-PCR

Primes for *SLC22A18* subcloning[1]	
Sense (EcoRI site)	5'-G**GA ATT C**CA TGC AGG GAG CTC GGG-3'
Antisense (XbaI site)	5'-GC**T CTA GA**T CAC CGG ACT TTG TCC TTC-3'
Primers for *SLC22A18* mutagenesis PCR[2]	
p.Ala6Thr (c.16G>A)	5'-CAG GGA GCT CGG **A**CT CCC AGG GAC C-3'
p.Arg12Gln (c.35G>A)	5'-GGA CCA GGG CC**A** GTC CCC CGG CA-3'
p.Arg86His (c.257G>A)	5'-CGC AGA CCA GC**A** CGG GGC GCG GG-3'
p.Trp324Cys (c.972G>C)	5'-GGC CAT GGC CTG **C**AT GTC CAG CGT C-3'
Primers for qRT-PCR	
SLC22A18 (sense)	5'- TAC CTG CAA ACC ACC TTC G-3'
SLC22A18 (antisense)	5'- AGC AGG TAG AGC GCC AAG-3'
GAPDH (sense)	5'- GTC TCC TCT GAC TTC AAC AGC G-3'
GAPDH (antisense)	5'- ACC ACC CTG TTG CTG TAG CCA A-3'

[1]Restriction endonuclease sites and [2]SNP sites are marked with bold-faced letters.
